# Improving sepsis care with Artificial Intelligence. What would Illich say?

**DOI:** 10.62675/2965-2774.20260261

**Published:** 2026-01-08

**Authors:** Matthieu Komorowski, Teresa Rincon, María del Pilar Arias Lopez, Rodrigo Octavio Deliberato, Leo Anthony Celi

**Affiliations:** 1 Charing Cross Hospital Intensive Care Unit Imperial College Healthcare NHS Trust London United Kingdom Intensive Care Unit, Charing Cross Hospital, Fulham Palace Road, Imperial College Healthcare NHS Trust - London, United Kingdom.; 2 Chingfen Graduate School of Nursing UMass Chan Medical School - Worcester Worcester MA United States Chingfen Graduate School of Nursing, UMass Chan Medical School - Worcester, MA, United States.; 3 Pediatric Intensive Care Unit Hospital de Niños Ricardo Gutierrez Buenos Aires Argentina Pediatric Intensive Care Unit, Hospital de Niños Ricardo Gutierrez - Buenos Aires, Argentina.; 4 University of Cincinnati College of Medicine Health Informatics and Data Science Department of Biostatistics Ohio United States The Translational Health Intelligence and Knowledge Lab, Department of Biostatistics, Health Informatics and Data Science, University of Cincinnati College of Medicine - Ohio, United States.; 5 Massachusetts Institute of Technology Laboratory for Computational Physiology Cambridge MA United States Laboratory for Computational Physiology, Massachusetts Institute of Technology -Cambridge, MA, United States.

## INTRODUCTION

Sepsis remains the leading cause of death in critical care and in hospitals. Artificial Intelligence (AI) is touted as having the potential to transform sepsis care through early recognition, personalized treatment, and enhanced decision-making. The need for these novel AI approaches is clearly endorsed by the Surviving Sepsis Campaign community, which recently produced a list of research priorities for sepsis, in an effort to move beyond "one-size-fits-all" approaches.^(1)^ These efforts center around the early recognition of sepsis, the characterization of organ dysfunction, and hemodynamic optimization.

However, improving outcomes through AI rests on a set of assumptions that deserve closer examination. In this Viewpoint, we interrogate those assumptions within the specific context of AI for sepsis prediction and management, through Illich's critique of medicalization. While the limits of aggressive care are familiar in critical-care ethics,^(2,3)^ they have not been systematically applied to AI in the intensive care unit (ICU) through the lens of Illich's philosophy. We aim to bridge these domains, propose a reframed, ethically grounded role for AI, and argue for a more fundamental shift in how sepsis is viewed for many patients.

## Assumptions behind the success of Artificial Intelligence in sepsis care

The success of AI in improving sepsis outcomes is underpinned by several beliefs and fundamental causal assumptions,^(4)^ such as:

- Sepsis is an acute, reversible disease which can be cured if appropriately managed.
- Early recognition of sepsis prevents the progression of organ dysfunction.^(5)^
- Personalized hemodynamic resuscitation will improve organ perfusion and function.^(6)^
- Data-driven methods can pin-point which patient needs a specific intervention at the right time.^(7)^

First and foremost, sepsis today is nearly always treated as an acute, reversible condition, with aggressive interventions such as antibiotics, vasopressors, mechanical ventilation, and invasive monitoring aimed at achieving physiological stability. Much of the research on AI-based sepsis prediction has focused on early detection, often prioritizing performance metrics such as Area Under the Receiver Operating Characteristic Curve (AUROC).^(4)^ Many of these studies are limited by data leakage, ambiguous definitions of sepsis and its onset, and validation strategies that overlook the complexities of real-world care.^(8,9)^ Yet for some patients, especially those with significant comorbidities, frailty, or advanced disease, sepsis may mark the natural end of life.^(3)^ Current AI models cannot distinguish sepsis as a potentially reversible condition from sepsis as part of the dying process, effectively predicting death rather than identifying truly treatable cases. Instead of focusing solely on reversing sepsis, clinicians should balance curative efforts with an acknowledgment of when such interventions might prolong suffering without meaningful benefit.

## Illich's views

These concepts were best laid out by the Austrian social critic and philosopher Ivan Illich, who expressed profound concerns about the industrialization of medicine, particularly regarding its impact on end-of-life care. His views on this subject are encapsulated in his influential works, such as his 1976's *Medical nemesis: the expropriation of health*.^(10)^

In this book, Illich argued that modern medicine had transformed death from a natural, communal experience into a medicalized, institutional process. By treating death as a failure of treatment, the system promotes aggressive, often futile interventions that prolong suffering and erode dignity. He denounced the displacement of personal autonomy and community responsibility and advocated reclaiming death from institutional control - restoring simplicity, autonomy, and meaning at the end of life.

However, introducing AI into end-of-life decision-making poses a paradox. In Illich's own terms, it risks an "algorithmic nemesis": a technological extension of the very institutional control he sought to resist. If AI is used in this domain at all, it must remain a subordinate tool in service of human judgment and shared decision-making, not a determinant of care pathways.

## What does this mean for sepsis treatment in 2026?

Many patients with sepsis die in ICUs after invasive, distressing procedures, often with insufficient attention to comfort and dignity.^(9)^ For patients identified as approaching life's end, Illich's principles support a shift from prolonging life at all costs toward comfort, dignity, and quality of life. Practically, this may mean earlier transition from aggressive treatment to palliative care, viewing sepsis as a prompt for timely goals-of-care discussions.

Hospital culture can marginalize families in decision-making, prioritizing technical expertise and protocols.^(3)^ A human-centered model requires genuine shared decision-making: transparent communication about the natural course of sepsis and realistic outcomes so patients and families can weigh the burdens and benefits of options.

Beyond a purely biomedical frame (lactate clearance, mean arterial pressure, organ scores), a biopsychosocial approach acknowledges emotional, spiritual, and cultural dimensions of dying.^(11)^ Interventions should be tailored to the individual rather than rigidly following protocols designed for survivable cases. We provide an overview of the core principles of the model of care we advocate in table 1.

**Table 1:** Contrasting the standard aggressive treatment of sepsis with a human-centered, Artificial Intelligence-enabled approach inspired by Illichian principles

Dimension	Standard aggressive sepsis treatment	Human-centered, AI-enabled approach
Philosophical foundation	Sepsis as a uniformly reversible acute disease	Sepsis as potentially a terminal event, particularly in patients with multimorbidity and frailty
Therapeutic goals	Maximize survival through physiological stabilization	Balance life prolongation with comfort, dignity, and quality of life
Intervention strategy	Protocolized: following the Surviving Sepsis Campaign	Individualized: symptom management, timely palliative care, minimal intervention when prognosis is poor
AI deployment focus	Identify early warning signs; optimize timing and type of curative intervention	Identify patients for whom sepsis is likely terminal *versus* reversible; support shared decision-making
Model of care	Biomedical, protocol-driven	Biopsychosocial, context-sensitive, and value-congruent
View of death	Death as treatment failure	Death as a natural part of life in certain trajectories
Role of families and patients	Limited; often marginalized in fast-paced, protocolized decision-making	Central; empowered to participate in defining goals of care and evaluating burdens of treatment
Healthcare culture	Industrialized, efficiency-driven	Reflective, relationship-based, attentive to the meaning and experience of dying
Ethical lens	Beneficence and non-maleficence narrowly defined as survival maximization	Autonomy, dignity, proportionality, and harm avoidance, including iatrogenic suffering
AI training paradigm	Outcome-labelling assumes reversibility; focuses on physiological improvement and performance metrics	Requires nuanced labels incorporating context, comorbidities, and end-of-life trajectories

AI - Artificial Intelligence.

## Reframing Artificial Intelligence's role

Concretely, we see two complementary uses for AI. First, where sepsis is plausibly reversible, high-quality early warning and treatment timing tools may improve outcomes.^(3-5)^ Second, where sepsis likely signals irreversible decline, AI could help identify indicators of limited benefit earlier and prompt timely goals-of-care conversations. This will require confronting many data problems in one place: labels that conflate dying with "failure," retrospective outcomes shaped by prior decisions (self-fulfilling prophecy),^(12)^ and historical datasets that assume reversibility, biasing models toward escalation. Could it be that what we call "self-fulfilling prophecy" in a negative sense represents clinicians correctly identifying patients who are dying? Transparent cohort construction, sensitivity analyses, and explicit labeling of uncertainty are therefore essential.

Human-centered AI should also evolve to integrate contextual data (such as patient biography, faith, advance directives, and stated treatment preferences) alongside clinical predictors. The goal is not to automate choices but to facilitate conversations about value-concordant care. Models should clearly communicate uncertainty, avoid overconfident outputs, and be designed to support, rather than supplant, clinician-patient-family deliberation. Rather than merely predicting survival or death, as prognostic tools already attempt, AI should help contextualize prognostic information within comorbidities, frailty, and patient goals to support ethically proportionate decision-making.

There is, inevitably, an ethical tension. If AI can meaningfully improve early recognition and treatment of reversible sepsis, withholding its use could deny patients a chance of survival. Conversely, deploying AI indiscriminately risks reinforcing over-treatment and prolonging suffering when reversibility is unlikely. Clinicians must navigate between the imperative to save lives through timely intervention and the obligation to avoid burdensome, non-beneficial treatment.

## Conclusion

We are not advocating "giving up" on trying to improve sepsis care. However, AI in sepsis will not deliver on its promises if we continue to always view sepsis as an intercurrent reversible event and sepsis deaths as a failure of treatment, rather than what often represents the end of life. Instead, we argue for a more nuanced understanding of the potential of AI in sepsis and a more holistic, patient-centered approach to sepsis care. AI needs to be humanized by humans.

## Data Availability

The contents are already available.

## References

[1] De Backer D, Deutschman CS, Hellman J, Myatra SN, Ostermann M, Prescott HC (2024). Surviving Sepsis Campaign Research Committee. Surviving sepsis campaign research priorities 2023. Crit Care Med.

[2] García Abejas A, Geraldes Santos D, Leite Costa F, Cordero Botejara A, Mota-Filipe H (2025). Salvador Vergés À. Ethical challenges and opportunities of AI in end-of-life palliative care: integrative review. Interact J Med Res.

[3] Manfredi RA, Trevino J, Yan F, Rahimi M, Shapiro E, Gharehdaghi P (2021). Early palliative intervention in septic patients reduces healthcare utilization. Am J Emerg Med.

[4] O’Reilly D, McGrath J, Martin-Loeches I (2023). Optimizing artificial intelligence in sepsis management: opportunities in the present and looking closely to the future. J Intensive Med.

[5] Adams R, Henry KE, Sridharan A, Soleimani H, Zhan A, Rawat N (2022). Prospective, multi-site study of patient outcomes after implementation of the TREWS machine learning-based early warning system for sepsis. Nat Med.

[6] Kattan E, Bakker J, Estenssoro E, Ospina-Tascón GA, Cavalcanti AB, Backer D (2022). Hemodynamic phenotype-based, capillary refill time-targeted resuscitation in early septic shock: The ANDROMEDA-SHOCK-2 Randomized Clinical Trial study protocol. Rev Bras Ter Intensiva.

[7] Kalimouttou A, Kennedy JN, Feng J, Singh H, Saria S, Angus DC (2025). Optimal vasopressin initiation in septic shock: The OVISS Reinforcement Learning Study. JAMA.

[8] Kamran F, Tjandra D, Heiler A, Virzi J, Singh K, King JE (2024). Evaluation of sepsis prediction models before onset of treatment. NEJM AI.

[9] Wang Z, Wang W, Sun C, Li J, Xie S, Xu J (2025). A methodological systematic review of validation and performance of sepsis real-time prediction models. NPJ Digit Med.

[10] Illich I (1976). Medical nemesis: the expropriation of health.

[11] Nelson JE, Bassett R, Boss RD, Brasel KJ, Campbell ML, Cortez TB (2010). Improve Palliative Care in the Intensive Care Unit Project. Models for structuring a clinical initiative to enhance palliative care in the intensive care unit: a report from the IPAL-ICU Project (Improving Palliative Care in the ICU). Crit Care Med.

[12] De-Arteaga M, Elmer J (2023;). Self-fulfilling prophecies and machine learning in resuscitation science. Resuscitation.

